# A Non-Touchscreen Tactile Wearable Interface as an Alternative to Touchscreen-Based Wearable Devices

**DOI:** 10.3390/s20051275

**Published:** 2020-02-26

**Authors:** Hyoseok Yoon, Se-Ho Park

**Affiliations:** 1 Division of Computer Engineering, Hanshin University, Gyeonggi-do, Osan-si 18101, Korea; 2 Korea Electronics Technology Institute, Mapo-gu, Seoul 03924, Korea; sehopark@keti.re.kr

**Keywords:** tactile sensors, touch sensors, human–machine interaction, wearable device, smartwatches, human-computer interaction, user interface

## Abstract

Current consumer wearable devices such as smartwatches mostly rely on touchscreen-based user interfaces. Even though touch-based user interfaces help smartphone users quickly adapt to wearable devices with touchscreens, there exist several limitations. In this paper, we propose a non-touchscreen tactile wearable interface as an alternative to touchscreens on wearable devices. We designed and implemented a joystick-integrated smartwatch prototype to demonstrate our non-touchscreen tactile wearable interface. We iteratively improved and updated our prototype to improve and polish interaction ideas and prototype integration. To show feasibility of our approach, we compared and contrasted form factors of our prototype against the latest nine commercial smartwatches in terms of their dimensions. We also show response time and accuracy of our wearable interface to discuss our rationale for an alternative and usable wearable UI. With the proposed tactile wearable user interface, we believe our approach may serve as a cohesive single interaction device to enable various cross-device interaction scenarios and applications.

## 1. Introduction

Wearable devices are gaining momentum as a promising post-smartphone platform. Wearables have several advantages over smartphones, they are smaller, lighter and most importantly can be worn on a user’s body. Yet, wearables fall short in other areas such as usability, user experience (UX), computation power, small screen and input modalities. Due to these limitations, smartphones are still dominating as a general-purpose platform while wearables particularly focus more on specialized areas such as healthcare, mining personal data (i.e., life-logging, quantified-self) [1], virtual reality (VR) and augmented reality (AR). Wearable devices need better human–computer interface/interaction (HCI) and human-machine interface/interaction (HMI) to surpass the success of its predecessor.

Most commercial wearable devices such as smartwatches, smart glasses and hearables have adopted touch-based user interfaces (UI) to operate them. Specifically, smartwatches have adopted touchscreen-based UI/UX similar to that of smartphones. Since smartphone users are already familiar with using touchscreens, they will easily get used to wearable devices with touchscreens as well. Even though, the familiarity with touchscreens works as an advantage, there also exists several limitations in different contexts of wearable devices. First, it is more difficult to accurately select target items on a small screen of a wearable device. Second, the touchscreen-based UIs require users’ full attention that conflicts with the freedom of wearability. Third, touchscreen-based UIs are unusable in certain circumstances such as when a user’s hands are wet or dirty. To overcome these limitations and challenges, HCI/HMI researchers are putting their efforts to introduce novel wearable interfaces and experiment with broader set of alternatives for wearable UIs. Currently, non-touchscreen tactile interaction is undervalued for wearable devices. We believe that there are promising opportunities in carefully orchestrated tactile interaction which can provide direct and intuitive ways for using wearable devices.

In this paper, we propose a non-touchscreen tactile wearable interface as an alternative to touchscreens on wearable devices. We designed and implemented a joystick-integrated smartwatch prototype to demonstrate our non-touchscreen tactile wearable interface. We iteratively improved and updated our prototype from separated components to an integrated smartwatch form factor. To show feasibility of our approach, we compared and contrasted form factors of our prototype against several commercial smartwatches in terms of their dimensions. We also show response time and accuracy of our wearable interface to discuss our rationale for an alternative and usable wearable UI.

## 2. Related Work

In this section, we review currently available UIs on commercial wearable devices. Then, we summarize recent attempts on developing novel wearable UIs in tactile interaction and other modality-based interactions.

### 2.1. User Interface on Commercial Wearable Devices

**Figure 1:**
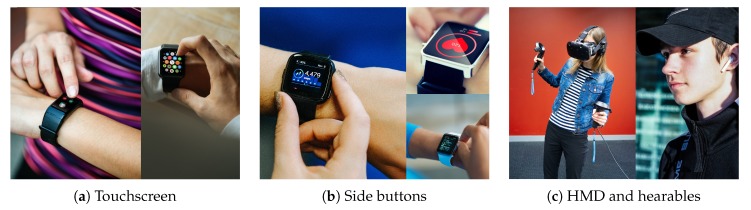
Typical wearable user interface modalities available on commercial products.

There are about four types of commercial wearable devices in the market. First type is wrist-worn wearables [2] such as smartwatches and smartbands. Second type is head-mounted displays (HMD) that is often used for VR entertainment and gaming. Third type is smart glasses such as Google Glass that is used for industrial applications and AR applications. Fourth type is hearables that users put on their ears for mostly audio applications. Figure 1 shows typical wearable UIs on commercial products.

- Smartwatches: Consumer smartwatches such as Apple Watch, Wear OS and Tizen-based smartwatches use touchscreen UIs. Additionally they provide side buttons such as digital crown and multi-function buttons to control without touching the touchscreen. Also voice-based interaction is provided through Apple’s Siri or Google Assistant. Nevertheless, the touchscreen UI is mostly used and preferred form of interaction for commercial smartwatches.
- HMD: HMDs such as Oculus Go and HTC Vive provide immersive VR experiences to users. Often they come with a separate controller for acquiring user inputs and sometimes natural hand gestures are recognized with computer vision.
- Smart glasses: Google glass and RealWear’s head mounted wearable provide see-through displays for industrial AR applications. These devices include side-touchpads and voice-based interaction is also supported.
- Hearables: Earables or hearables or wireless headset/earphones have recently gained popularity for audio applications. They often provide a touch-enabled control (i.e., single touch and double touch) in their small form factor.

### 2.2. Tactile Interaction

Tactile interaction contributes to enhance HCI/HMI in addition to visual and audio information/feedback in interactive systems. In using wearable devices, users can manipulate the device through a combination of movement and touch. Since wearable devices have small touchscreens for interaction, non-touchscreen tactile interaction becomes more important. As discussed in [3], tactile perception, kinesthetic perception and haptic perception should be considered in designing tactile interaction accordingly. Within context of wearable devices, there are two groups of work in tactile interaction. One group of work employs touchscreen and other group of work explores non-touchscreen, yet tactile interaction for wearables.

Touchscreen-Driven Interaction. There are many studies that aim to use small touchscreens on wearable devices more efficiently such as Beats [4] and WristTap & TwoTap techniques [5]. Specifically, an area of interest for wearable computing researchers is to improve touchscreen-based soft keyboards for text entry. SplitBoard purposely divided the wide QWERTY layout into left and right sections to deal with small keys on a smartwatch [6]. Leiva et al. developed a callout-based soft keyboard and ZShift to assist text entry on small screens [7].

Non-Touchscreen Interaction. Other than touchscreens, different parts of wearable devices are turned into touch-sensitive input modules. Funk et al. developed a touch-sensitive wristband prototype with a potentiometer [8]. Similarly, BandSense allowed pressure-sensitive multi-touch interaction on a wristband [9] and N-ary input demonstrated a text entry application using force-sensitive linear potentiometers (FSLP) [10]. EdgeTouch prototype used capacitive sensors embedded on the edges of the prototype to sense user-interaction [11]. Yoon et al. developed lightful user interaction on smart wearables that exploit ambient light sensor as touch sensors [12] and DeLightTouch as an extended multi-touch input method [13]. Gong et al. developed Cito where an actuated smartwatch face was used to make movements such as rotation, hinging, translation, rising and orbiting for interaction events [14]. In Indutivo, a contact-based inductive sensing was implemented to recognize various conductive objects for interaction [15]. Seyed et al. presented Doppio that reconfigured two touch sensitive display faces to create tangible inputs [16]. Pakanen et al. developed squeeze based tactile interaction techniques on a bracelet form factor [17]. Xiao et al. developed a prototype to support pan, twist, tilt, click movements with the watch face employing joystick sensors [18].

Several studies expanded UI space to the user’s skin. SkinButtons integrated a tiny projector into the smartwatch where touch-sensitive icons were projected onto the user’s skin [19]. Xiao et al. presented LumiWatch as a fully functional projection smartwatch that enabled touch input on the skin [20]. Lim et al. expanded smartwatch touch interface to the back of the user’s hand using infrared (IR) line image sensors [21]. Lee et al. developed a machine learning based side-tap recognition using the built-in 9-axis motion sensors (accelerometer, gyroscope and linear acceleration) [22].

Other Modality-based Interaction. Recently, different modalities are explored in wearable UIs. Notable research directions include gesture-based UIs that use sensor data and machine learning techniques. Yu et al. developed motion UI using the smartwatch’s built-in 6-axis sensors to identify four directional inputs [23]. Kwon et al. developed the convolution neural network (CNN) based gesture pattern recognition using an accelerometer sensor of a smartwatch [24]. Laput el al. also developed CNN-based hand activity classifier for sensing fine-grained hand activities with the smartwatch’s three-axis accelerometer data [25]. Yeo et al. developed WRIST with a sensor fusion approach to combine inertial measurement unit (IMU) data from a smartwatch and a smart ring [26]. For a concise summary on different wearable UIs, interested readers are referred to [27].

Among the three aforementioned groups of work, we focus on designing and implementing non-touchscreen tactile interaction for wearables. Our attempts for implementing alternative tactile wearable UI are described in following Section 3.

## 3. Alternative Tactile Wearable User Interface

We believe that wearable devices have great potential for becoming the next main computing platform. However, most wearable UIs are at the stage of recreating trite touchscreen-based interaction that hold back a breakthrough and limit various applications in wearable UIs. Therefore, HCI/HMI researchers should explore and invent novel wearable UIs beyond the touchscreen-based UIs. In this section, we identify problems and issues of using touchscreens on wearable devices and argue for the needs of alternative and optional wearable UIs.

### 3.1. Touchscreens on Wearable Device

The mainstream wearable user interface is arguably touchscreen. Smartphone-like UI/UX is attractive to end-users and therefore easily applied to consumer wearable devices such as smartwatches. Touchscreens on smartwatches have several advantages indeed. Since the device’s touchscreen is used for both input and output purposes, it only requires a small footprint to realize the device in a wristwatch form factor. Also it does not introduce additional technical barrier to end-users who have previous experience on smartphones. On the other side, there are some disadvantages and limitations on using touchscreens on wearable devices. The user has to be very attentive to use touchscreen (i.e., look at the screen while interacting). It is difficult to use a small touchscreen when several items or lists are displayed. Because the touchscreen is used for both input and output purposes, only a small amount of information is displayed and much of the touchscreen is blocked with the user’s fingers. Also touchscreen does not work well with contaminated fingers (i.e., wet and dirty) leaving smudges afterwards and resulting inaccurate selections.

### 3.2. Rationale and Design Consideration

As briefly discussed in the previous subsection, touchscreen is intuitive yet there exists several limitations. Are there alternative interaction methods that have advantages of tactile interaction while maintaining a small form factor? If we can achieve alternative wearable UIs, then end-users will be given many options to choose from. We have addressed other approaches such as using side buttons (i.e., multi-function buttons and digital crown), potentiometer sensors and rotating bezels as examples of non-touchscreen tactile wearable UIs. We considered 3 requirements for designing and implementing an alternative tactile wearable user interface that can complement or for some cases replace the touchscreen-based UIs.

1. Form factor: self-contained in a wearable device. The proposed alternative wearable UI should be self-contained within the device and should not make the device form factor unnecessarily larger. To elaborate, alternative UI should not make the wearable device un-wearable or bulky.
2. Input modality: non-occluding, tactile, direct and intuitive. The proposed alternative wearable UI should be tactile, direct and intuitive for enhanced usability and applicability. The alternative UI should be usable for most common wearable applications.
3. Input events: expressive, responsive and accurate. The proposed alternative wearable UI should enable expressive, responsive and accurate input events to be generated, gathered and interpreted.

## 4. Iterative Prototyping of Joystick-Based Wearable User Interface

To design and implement an alternative tactile wearable UI, we iteratively improved and updated our prototype over time. For tactile wearable UI, we specifically focused on contact-based UI with a joystick sensor. Previously joysticks are mostly used in gaming context for selecting certain targets using a game controller analog thumbstick [28]. Other uses of joysticks include issuing driving commands for wheelchairs [29] and text entry in VR systems [30]. Usability and UX aspects of the joystick-embedded UI and other wearable UIs are thoroughly examined in [27]. In this paper, we report on consecutive and incremental ideas that resulted in our intermediate and final prototypes.

### 4.1. Thumbstick-Based Interaction

**Figure 2:**
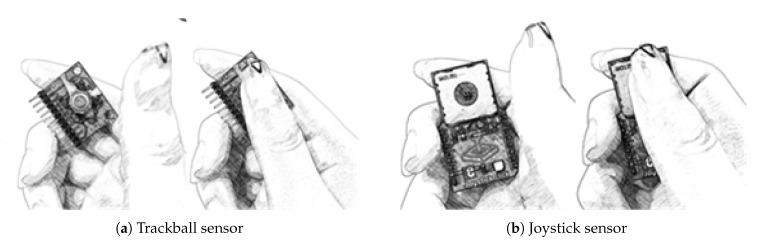
Exploration of thumbstick-based interaction with small tactile sensor candidates.

As mentioned in our rationale and design consideration, we wanted to design and develop a wearable UI that can be self-contained in a wearable device. For this reason, a small tactile sensor is considered. We explored with two sensors, a trackball sensor and a joystick sensor for thumbstick-based interaction as shown in Figure 2.

- Trackball sensor. A trackball sensor module includes a small ball as a pointing device as shown in Figure 2a. When the user rotates this ball, relative movement such as up, down, left and right direction can be measured. This module is small, so it can be attached or integrated to the small form factor of a wearable device.
- Joystick sensor. A joystick sensor module includes a small stick that can be manipulated to indicate direction movements as shown in Figure 2b. This module is also small and can be easily attached or integrated to the small form factor of a wearable device.

Both trackball and joystick meet the first requirement (i.e., self-contained in a wearable device form factor). As an application for wearable device, text entry methods using a trackball sensor and a joystick sensor were implemented [31,32]. From two sensors, we ended up selecting the joystick sensor because it is more tactile, direct and intuitive. When the joystick is manipulated, the user can hear a sound of clicking and make more direct input even without looking at the sensor module. Furthermore, the joystick sensor module is able to produce repetitive and continuous input events better than the trackball sensor module. For example, it is cumbersome to input 10 left input events with the trackball sensor by moving the trackball back and forth repetitively. With the joystick module, after one left input event is fired, the joystick can be stayed at the same position to continuously fire consecutive input events.

### 4.2. First Prototype: An Initial Proof of Concept for Joystick-Based Interaction

**Figure 3:**
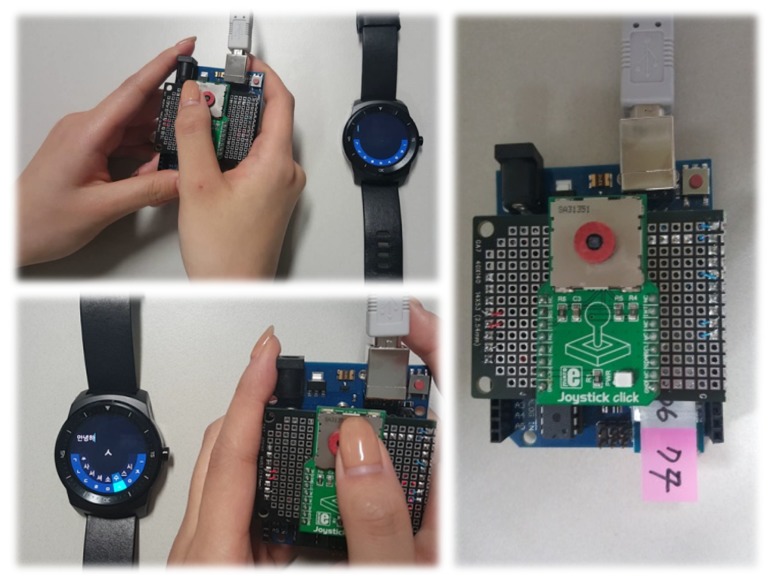
First prototype with a separated joystick control.

We implemented a proof of concept (PoC) with Arduino and a joystick sensor. In the first prototype, joystick movements or events are delivered to a paired smartwatch via Bluetooth Serial Port Profile (SPP). Regarding latency and input expressivity (4 direction inputs, left, right, up and down and a click event) of the joystick-based interaction, we found it as a promising alternative tactile wearable UI. Various combination of input events were expressive enough for simple menu navigation and for more complex text entry (i.e., Korean text input). Figure 3 shows our first prototype with a separated joystick control.

### 4.3. Whole Device Interaction

**Figure 4:**
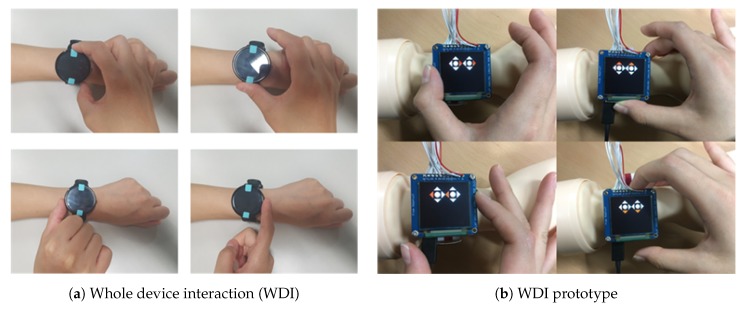
Exploration of thumbstick-based interaction.

Even though we witnessed the joystick sensor module as a new additional input modality in the first prototype, the device and the sensor module were not physically integrated. Therefore, the total footprint of the first prototype was larger than most commercial smartwatch counterparts. To improve, we considered various ways to integrate the joystick sensor and the device together. Inspired by [18], we put the joystick sensor right beneath the screen. So when the screen is moved, the joystick beneath the screen also moved to create input events. We refer to this concept as whole device interaction (WDI) where the user touches and moves the smartwatch by exerting forces sideways, upward and downward as shown in Figure 4a. Figure 4b shows WDI after the physical integration.

### 4.4. Second Prototype: Physical Integration

**Figure 5:**
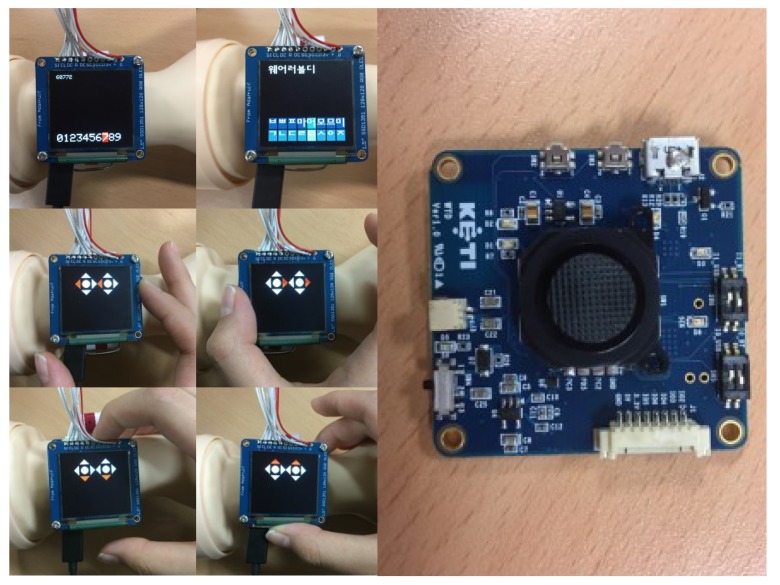
Second prototype integrated with a joystick and a screen.

Our second prototype brought together the separated joystick control and the screen (i.e., not a touchscreen). For the second prototype iteration, we focused on creating an alternative wearable UI targeting smartwatches or wrist-worn wearables. So we build up a functional smartwatch mock-up and explored joystick-based interaction. Unlike our first idea of using a joystick as a thumbstick interface, we found that using multiple fingers to manipulate the whole device was more natural and intuitive. The second prototype physically integrated the screen and the joystick using two boards stocked up on top of one another. To test the second prototype’s minimal functionality, we developed several apps for direction input and number/text entry purposes as shown in Figure 5.

### 4.5. Final Prototype: Enclosed in a Smartwatch Form Factor

**Figure 6:**
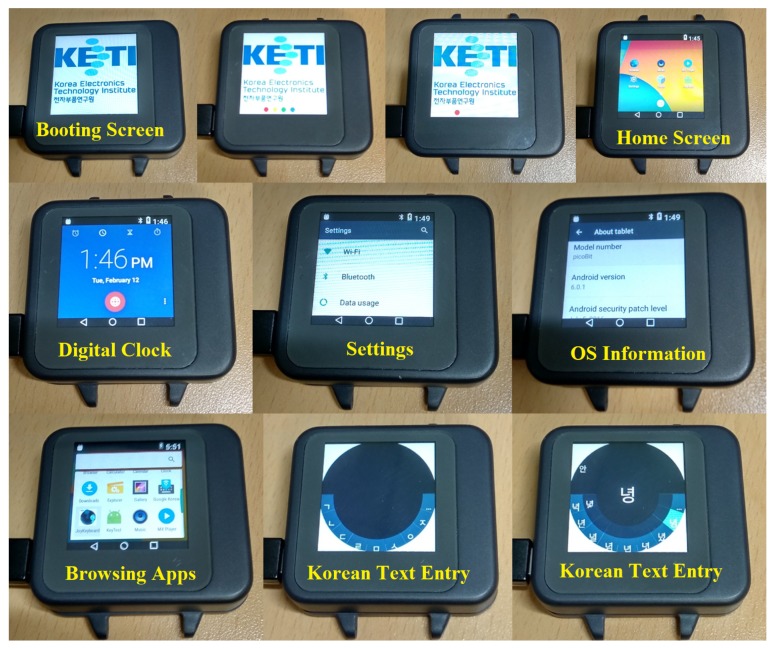
Final prototype with an enclosed case to resemble a smartwatch form factor.

To make our prototype fully functional and inter-operable, we chose Android OS and also added Bluetooth/WiFi. We specifically focused on developing a prototype that resembled a smartwatch form factor. Figure 6 shows the final prototype with an enclosed case that can be worn on a user’s wrist. Since our prototype runs Android OS version 6.0.1, Android apps can be executed on this device as in other Android-based tablets and smartphones. After the booting screen, only icons for selected apps are displayed in the home screen while more apps can be browsed. To manipulate this home screen, our joystick input is used to navigate among app icons and click to select an app to launch. Furthermore, a digital clock displays current time and dates as in other commercial smartwatches. Advanced settings menus for Wi-Fi, Bluetooth and OS information can be accessed in Settings. We have developed an App for Korean text entry to specifically test four directional inputs and click input provided by our prototype.

**Figure 7:**
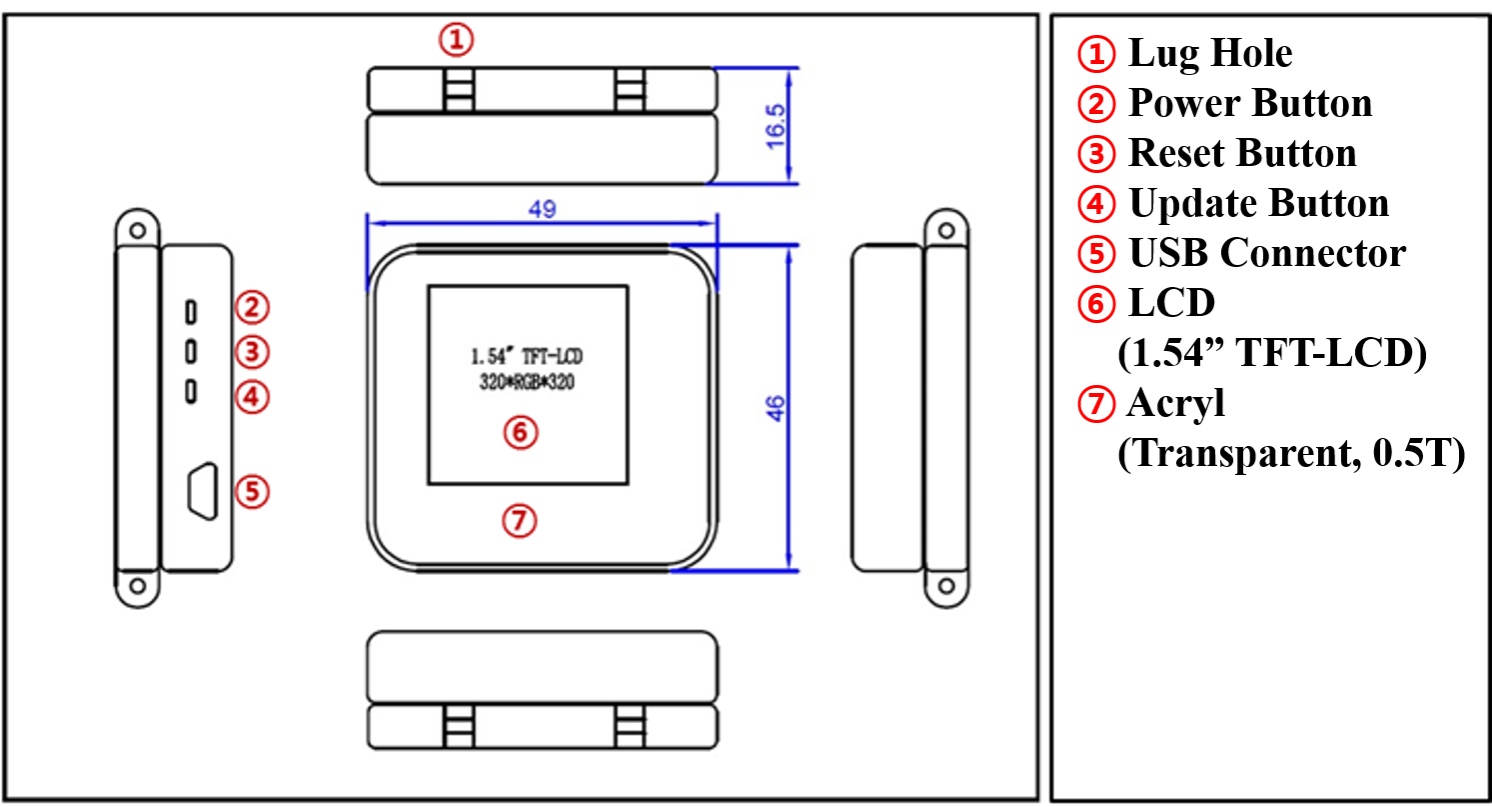
A blueprint for the final prototype with parts description.

Figure 7 shows a blueprint for the final prototype. The final product had lug holes so strap or bands can be attached as in commercial smartwatches. Three side buttons and USB connector were added to make testing and developing test apps convenient. All parts were carefully selected and designed so that the size of the final product does not exceed sizes of comparable commercial smartwatches. Table 1 shows detailed specification of the final prototype.

## 5. Experiments

In this section, we show feasibility of our non-touchscreen tactile wearable UI in two criteria. First, we show that our wearable UI can be fully realized while conforming to the standard smartwatch size in commercial products. Second, we show that our wearable UI produces responsive and accurate input events appropriate for basic wearable applications.

### 5.1. Form Factor

**Figure 8:**
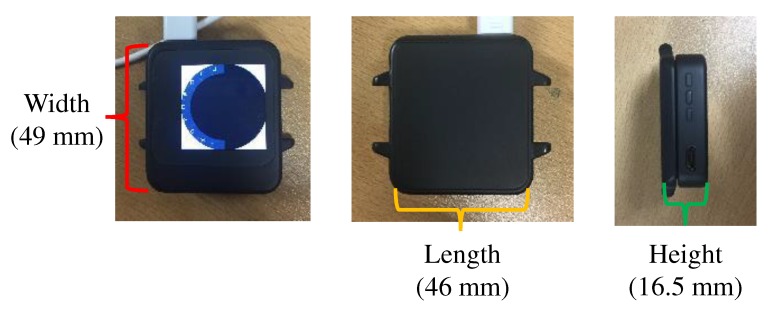
The final prototype measures 49 × 46 × 16.5 mm.

**Figure 9:**
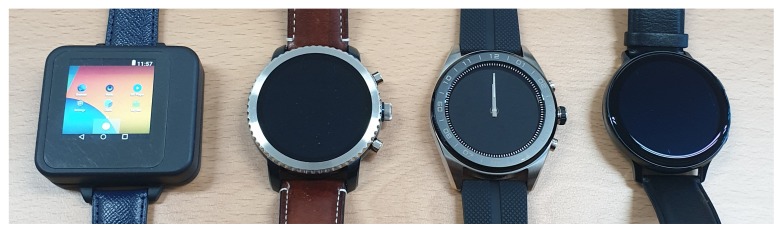
A side-by-side size comparison with commercial smartwatches (depicted from left to right, our prototype, Fossil Gen 3, LG Watch W7 and Samsung Galaxy Watch Active2, respectively).

**Figure 10:**
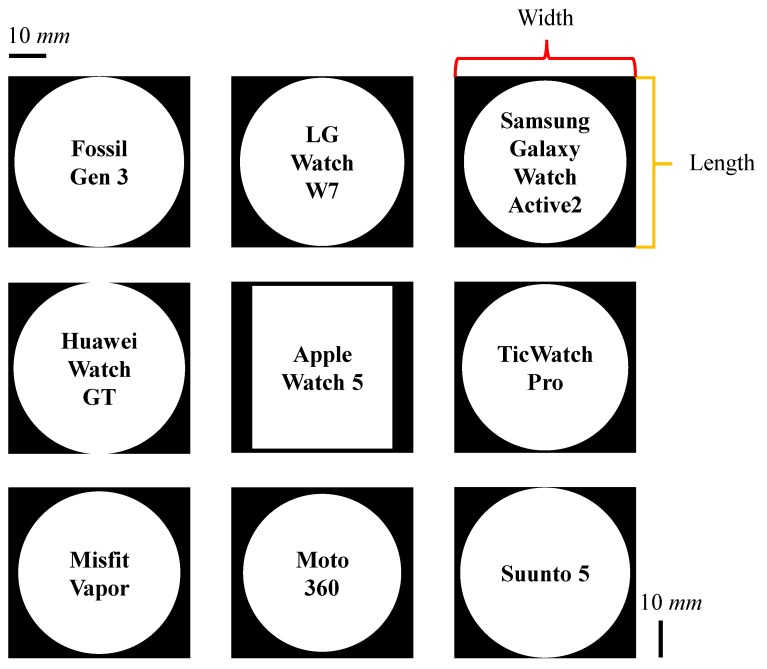
Visual comparisons and contrasts of width × length. Outer black boxes represent the size of our final prototype. Refer to the included scale bar for actual dimension comparison.

**Figure 11:**
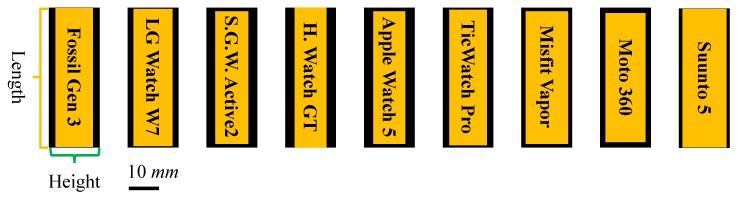
Visual comparisons and contrasts of length × height (thickness). Outer black boxes represent the size of our final prototype. Refer to the included scale bar for actual dimension comparison.

Our joystick-embedded, non-touchscreen and wearable device prototype was designed and implemented to resemble commercial smartwatches. The dimension of the final prototype was 49 × 46 × 16.5 mm as shown in Figure 8. Figure 9 shows a side-by-side size comparison with three commercial smartwatches visually. Most commercial smartwatches have a round watch face and have one or more side buttons or multi-function buttons. Table 2 lists dimensions of additional latest smartwatches and our prototype. Our prototype is indeed relatively thicker than other smartwatches, but otherwise comparable to other commercial products. To show our prototype is relatively competitive in sizes, we present Figure 10 and Figure 11 for visual comparison and contrast. One technical difficulty we encountered was finding a round shape display when we first implemented the prototypes. So we ended up implementing a rectangular shaped smartwatch which was not a typical and recent choice for smartwatch makers, other than Apple. Overall, we have successfully produced our alternative tactile wearable UI in a smartwatch size.

### 5.2. Interface Response Speed and Interface Control Accuracy

In user interface, it is important to make inputs reasonably fast, responsive and accurate. To show feasibility of our prototype as a viable wearable UI, we measured response time and accuracy of input events. Response time refers to the total time (sensing time + time for event recognition). We repetitively made 100 input events for four directions (i.e., left, right, up and down events) with our prototype and measured how long it took per an input event. To measure response time, we used Android Monitor Profiling Tools to log starting time, end time, number of input events and average time. Table 3 shows the summary of the tested input events. It took 55–61 ms to input and recognize an event by the system. Accuracy of all four events were 100%, meaning that no input event is wrongly interpreted as another event. To elaborate, while entering 100 input events, they were all correctly identified and never falsely identified as some other events. This type of wearable UI has advantages over gesture recognition-based UIs (i.e., classifiers in machine learning [22,24,25,33]), since input events can be falsely identified as incorrect events to be triggered.

**Figure 12:**
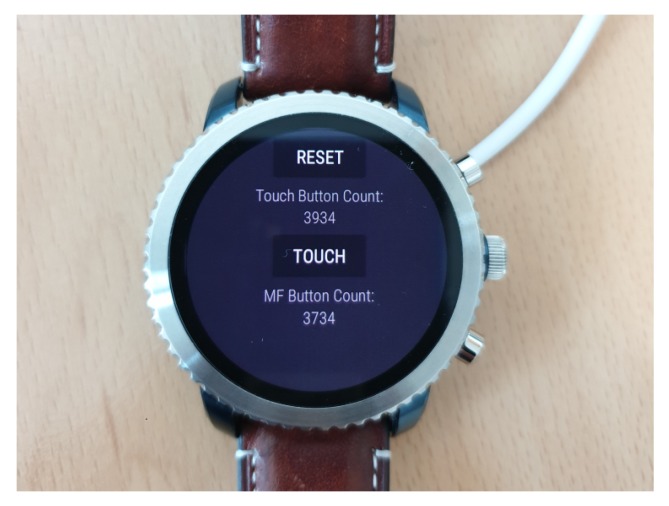
Traditional UI response time measured on commercial Wear OS smartwatch (Fossil Gen 3).

To compare response time with traditional UI found on commercial smartwatches, we developed a sample app on a Wear OS smartwatch as shown in Figure 12. In this app, we measured response times for a touchscreen button and a multi-function button. We repetitively generated 100 click events to calculate average response times for two UI found on commercial smartwatches. Also in [23], response time for Android Wear gestures was reported between 700 to 1200 ms. We provide Table 4 to compare performance of our non-touchscreen tactile UI against commercial systems. Our UI does not require much computation compare to [23]. For basic applications, our UI is as responsive as touchscreen button and multi-function button. For cases when wearable devices lack touchscreen or side-buttons, alternative wearable UI such as our proposed UI can complement the limited input capability.

### 5.3. Application

**Figure 13:**
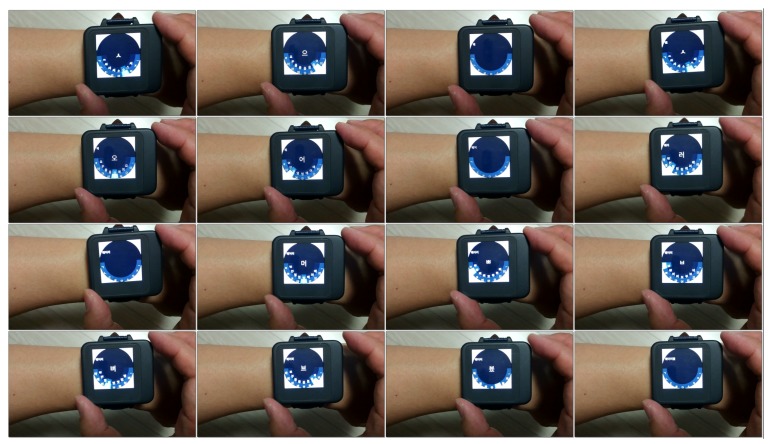
Korean text entry application on the final prototype.

Figure 13 shows a Korean text entry application [31,32] running on the final prototype. Four directional events are created by moving the smartwatch face left, right, up and down. Then these events are mapped as KeyEvent in Android OS such as KEYCODE_DPAD_LEFT, KEYCODE_DPAD_RIGHT, KEYCODE_DPAD_UP and KEYCODE_DAPD_DOWN, respectively, for internal application use.

### 5.4. Limitations

In this paper, our work focused on the physicality of the wearable prototype and physical sensing aspects. To demonstrate these aspects, we compared physical dimensions and sensing capability (i.e., response time) of basic operations with commercial devices. Nonetheless, we do believe that assessing usability and user experience aspects including task completion assessment are important. For example, in our study on wearable UI evaluation framework [27], we specifically evaluated usability principles and assessed task workload with a primitive version of our prototype along with other novel wearable UI prototypes. Even though an earlier version of our wearable tactile prototype is used, we have witnessed feasibility of our approach as for both alternative and complementary user interface. Since usability evaluation of our integrated wearable prototype is beyond the scope of this paper, we acknowledge that this topic deserves further studies along with designing, developing and deploying various tasks and applications under specific scenarios.

## 6. Conclusions

In this paper, we proposed a non-touchscreen tactile wearable UI by integrating a joystick into the smartwatch form factor. To improve and enhance prototypes, we iteratively updated our prototypes from separated modules to a single integrated smartwatch. We also changed our UI technique from thumbstick interaction (i.e., using one finger) to WDI (i.e., use multiple fingers to move the whole device). We carefully designed and implemented our prototype so that it conforms to the typical smartwatch size. In our experiments, we showed that our prototype is competitive with commercial smartwatches in its size while input events are generated responsively (55–61 ms) and accurately. We believe our approach can be useful in VR entertainment and games scenarios for using the smartwatch as an effective controller when coupled with an HMD [30,33,34]. Especially for a cross-device ecology where multiple devices are seamlessly connected [35], our approach may serve as a cohesive single interaction device to enable various cross-device interaction scenarios and applications at different scales for near, personal, social and public levels [36]. 

## Figures and Tables

**Table 1:** Final prototype specification.

Components	Specification
Microcontroller Unit (MCU)	RK3128 Quad-core Cortex-A7 (Max 1.2 GHz)
USB Standards	USB 2.0 OTG/Host
Power	DC 5V
Operating Environment	−5° to 40°
Memory	EMMC 4 GByte, LPDDR2 4 Gbit
Operating System	Android OS 6.0.1
Functions	Up, Down, Left, Right, Click
Display	TFT LCD 320 × 320
Bluetooth	Bluetooth 4.0
WiFi	2.4G/5G a/b/g/n
Size	49 × 46 × 16.5 mm

**Table 2:** Dimension comparison with commercial smartwatches. The largest size is denoted with a symbol ^†^ followed by the second largest with a symbol ${}^{‡}$.

Smartwatch Model	Width (mm)	Length (mm)	Height or Thickness (mm)
Our Prototype	**49 ${}^{†}$**	**46 ${}^{‡}$**	**16.5 ${}^{†}$**
Fossil Gen 3	46	**46 ${}^{‡}$**	12.5
LG Watch W7	44.5	45.5	13
Samsung Galaxy Watch Active2	44	44	10.9
Huawei Watch GT	**46.5 ${}^{‡}$**	**46.5 ${}^{†}$**	10.6
Apple Watch 5	38	44	10.7
TicWatch Pro	45	45	12.6
Misfit Vapor	44	44	14
Moto 360	42.8	42.8	13.28
Suunto 5	46	**46 ${}^{‡}$**	**14.6 ${}^{‡}$**

**Table 3:** Experimental results for 4 directional events.

	Left Event	Right Event	Up Event	Down Event
Response Time (ms)	55	55	61	58
Accuracy (out of 100 repetitions)	100%	100%	100%	100%

**Table 4:** Response times for different UIs available on commercial smartwatches.

	Touchscreen Button	Multi-Function Button	Wear Gesture API [23]
Response Time (ms)	39	37	about 700 to 1200

## Abbreviations

The following abbreviations are used in this manuscript:

UX	User eXperience
VR	Virtual Reality
AR	Augmented Reality
HCI	Human-Computer Interface or Interaction
HMI	Human-Machine Interface or Interaction
FSLP	Force-Sensitive Linear Potentiometer
UI	User Interface
HMD	Head Mounted Display
IR	Infrared Radiation
IMU	Inertial Measurement Unit
CNN	Convolution Neural Network
PoC	Proof of Concept
SPP	Serial Port Profile
WDI	Whole Device Interaction
MCU	Microcontroller Unit
OTG	USB On-The-Go

## References

[1] Rehman M., Liew C., Wah T., Shuja J., Daghighi B. (2015). Mining Personal Data Using Smartphones and Wearable Devices: A Survey. Sensors.

[2] Kamišalić A., Fister I., Turkanović M., Karakatič S. (2018). Sensors and Functionalities of Non-Invasive Wrist-Wearable Devices: A Review. Sensors.

[3] Challis B. (2013). The Encyclopedia of Human-Computer Interaction.

[4] Oakley I., Lee D., Islam M.R., Esteves A. Beats: Tapping Gestures for Smart Watches. Proceedings of the 33rd Annual ACM Conference on Human Factors in Computing Systems.

[5] Lafreniere B., Gutwin C., Cockburn A., Grossman T. Faster Command Selection on Touchscreen Watches. Proceedings of the 2016 CHI Conference on Human Factors in Computing Systems.

[6] Hong J., Heo S., Isokoski P., Lee G. SplitBoard: A Simple Split Soft Keyboard for Wristwatch-sized Touch Screens. Proceedings of the 33rd Annual ACM Conference on Human Factors in Computing Systems.

[7] Leiva L.A., Sahami A., Catala A., Henze N., Schmidt A. Text Entry on Tiny QWERTY Soft Keyboards. Proceedings of the 33rd Annual ACM Conference on Human Factors in Computing Systems.

[8] Funk M., Sahami A., Henze N., Schmidt A. Using a touch-sensitive wristband for text entry on smart watches. Proceedings of the extended abstracts of the 32nd annual ACM conference on Human factors in computing systems.

[9] Ahn Y., Hwang S., Yoon H., Gim J., Hee Ryu J. BandSense: Pressure-sensitive Multi-touch Interaction on a Wristband. Proceedings of the 33rd Annual ACM Conference Extended Abstracts on Human Factors in Computing Systems.

[10] Yoon H., Lee J.E., Park S.H., Lee K.T. Position and Force Sensitive N-Ary User Interface Framework for Wrist-Worn Wearables. Proceedings of the 2016 Joint 8th International Conference on Soft Computing and Intelligent Systems and 17th International Symposium on Advanced Intelligent Systems.

[11] Oakley I., Lee D. Interaction on the edge: Offset sensing for small devices. Proceedings of the 32nd annual ACM conference on Human factors in computing systems.

[12] Yoon H., Park S.H., Lee K.T. (2016). Lightful user interaction on smart wearables. Pers. Ubiquit. Comput..

[13] Yoon H., Park S.H., Lee K.T. DeLightTouch: Light sensor assisted multi-touch gestures on unmodified commodity smartwatches. Proceedings of the 2017 International Conference on Information and Communication Technology Convergence.

[14] Gong J., Li L., Vogel D., Yang X.D. Cito: An Actuated Smartwatch for Extended Interactions. Proceedings of the 2017 CHI Conference on Human Factors in Computing Systems.

[15] Gong J., Yang X., Seyed T., Davis J.U., Yang X.D. Indutivo: Contact-Based, Object-Driven Interactions with Inductive Sensing. Proceedings of the 31st Annual ACM Symposium on User Interface Software and Technology.

[16] Seyed T., Yang X.D., Vogel D. Doppio: A Reconfigurable Dual-Face Smartwatch for Tangible Interaction. Proceedings of the 2016 CHI Conference on Human Factors in Computing Systems.

[17] Pakanen M., Colley A., Häkkilä J., Kildal J., Lantz V. Squeezy bracelet: Designing a wearable communication device for tactile interaction. Proceedings of the 8th Nordic Conference on Human-Computer Interaction Fun, Fast, Foundational.

[18] Xiao R., Laput G., Harrison C. Expanding the input expressivity of smartwatches with mechanical pan, twist, tilt and click. Proceedings of the 32nd Annual ACM Conference on Human Factors in Computing Systems.

[19] Laput G., Xiao R., Chen X.A., Hudson S.E., Harrison C. Skin buttons: Cheap, small, low-powered and clickable fixed-icon laser projectors. Proceedings of the 27th Annual ACM Symposium on User Interface Software and Technology.

[20] Xiao R., Cao T., Guo N., Zhuo J., Zhang Y., Harrison C. LumiWatch: On-Arm Projected Graphics and Touch Input. Proceedings of the 2018 CHI Conference on Human Factors in Computing Systems.

[21] Lim S.C., Shin J., Kim S.C., Park J. (2015). Expansion of Smartwatch Touch Interface from Touchscreen to Around Device Interface Using Infrared Line Image Sensors. Sensors.

[22] Lee K.T., Yoon H., Lee Y.S. Implementation of smartwatch user interface using machine learning based motion recognition. Proceedings of the 2018 International Conference on Information Networking.

[23] Yu S.B., Yoon H., Park S.H., Lee K.T. Motion UI: Motion-based user interface for movable wrist-worn devices. Proceedings of the 2017 IEEE 7th International Conference on Consumer Electronics.

[24] Kwon M.C., Park G., Choi S. (2018). Smartwatch User Interface Implementation Using CNN-Based Gesture Pattern Recognition. Sensors.

[25] Laput G., Harrison C. Sensing Fine-Grained Hand Activity with Smartwatches. Proceedings of the 2019 CHI Conference on Human Factors in Computing Systems.

[26] Yeo H.S., Lee J., Kim H., Gupta A., Bianchi A., Vogel D., Koike H., Woo W., Quigley A. WRIST: Watch-Ring Interaction and Sensing Technique for Wrist Gestures and Macro-Micro Pointing. Proceedings of the 21st International Conference on Human-Computer Interaction with Mobile Devices and Services.

[27] Yoon H., Park S.H., Lee K.T., Park J., Dey A., Kim S. (2017). A Case Study on Iteratively Assessing and Enhancing Wearable User Interface Prototypes. Symmetry.

[28] Claypool M. (2018). Game Input with Delay—Moving Target Selection with a Game Controller Thumbstick. ACM Trans. Multim. Comput..

[29] Rofer T., Mandel C., Laue T. Controlling an automated wheelchair via joystick/head-joystick supported by smart driving assistance. Proceedings of the 2009 IEEE International Conference on Rehabilitation Robotics.

[30] Yu D., Fan K., Zhang H., Monteiro D., Xu W., Liang H.N. (2018). PizzaText: Text Entry for Virtual Reality Systems Using Dual Thumbsticks. IEEE Trans. Visual Comput. Graph..

[31] Lee J.E., Ahn J.E., Park K., Choi G.E., Moon I.Y. (2015). Design and Implementation of Trackball Based UI for Efficient Text Entry on Smartwatch. J. Adv. Nav. Tech..

[32] Lee J.E., Yoon H., Park S.H., Lee K.T. Complete as you go: A constructive Korean text entry method for smartwatches. Proceedings of the 2016 International Conference on Information and Communication Technology Convergence.

[33] Yoon H., Sung J.Y., Park S.H. Re-targeting User Interfaces on Stand-alone Smart Wearables. Proceedings of the 2019 International Conference on Information and Communication Technology Convergence.

[34] Kim H.I., Woo W. Smartwatch-assisted robust 6-DOF hand tracker for object manipulation in HMD-based augmented reality. Proceedings of the 2016 IEEE Symposium on 3D User Interfaces (3DUI).

[35] Houben S., Marquardt N., Vermeulen J., Klokmose C., Schöning J., Reiterer H., Holz C. (2017). Opportunities and Challenges for Cross-device Interactions in the Wild. Interactions.

[36] Brudy F., Holz C., Rädle R., Wu C.J., Houben S., Klokmose C.N., Marquardt N. Cross-Device Taxonomy: Survey, Opportunities and Challenges of Interactions Spanning Across Multiple Devices. Proceedings of the 2019 CHI Conference on Human Factors in Computing Systems.

